# Intensive post-operative follow-up of breast cancer patients with tumour markers: CEA, TPA or CA15.3 vs MCA and MCA-CA15.3 vs CEA-TPA-CA15.3 panel in the early detection of distant metastases

**DOI:** 10.1186/1471-2407-6-269

**Published:** 2006-11-20

**Authors:** Andrea Nicolini, Gianna Tartarelli, Angelo Carpi, Maria Rita Metelli, Paola Ferrari, Loretta Anselmi, Massimo Conte, Piero Berti, Paolo Miccoli

**Affiliations:** 1 Department of Internal Medicine, University of Pisa, Pisa, Italy; 2 Department of Reproduction and Ageing, University of Pisa, Pisa, Italy; 3 Department of Experimental Pathology, University of Pisa, Pisa, Italy; 4 Department of Surgery, University of Pisa, Pisa, Italy

## Abstract

**Background:**

In breast cancer current guidelines do not recommend the routine use of serum tumour markers. Differently, we observed that CEA-TPA-CA15.3 (carcinoembryonic (CEA) tissue polypeptide (TPA) and cancer associated 115D8/DF3 (CA15.3) antigens) panel permits early detection and treatment for most relapsing patients. As high sensitivity and specificity and different cut-off values have been reported for mucin-like carcinoma associated antigen (MCA), we compared MCA with the above mentioned tumour markers and MCA-CA15.3 with the CEA-TPA-CA15.3 panel.

**Methods:**

In 289 breast cancer patients submitted to an intensive post-operative follow-up with tumour markers, we compared MCA (cut-off values, ≥ 11 and ≥ 15 U/mL) with CEA or CA15.3 or TPA for detection of relapse. In addition, we compared the MCA-CA15.3 and CEA-TPA-CA15.3 tumour marker panels.

**Results:**

Distant metastases occurred 19 times in 18 (6.7%) of the 268 patients who were disease-free at the beginning of the study. MCA sensitivity with both cut-off values was higher than that of CEA or TPA or CA15.3 (68% vs 10%, 26%, 32% and 53% vs 16%, 42%, 32% respectively). With cut-off ≥ 11 U/mL, MCA showed the lowest specificity (42%); with cut-off ≥ 15 U/mL, MCA specificity was similar to TPA (73% vs 72%) and lower than that of CEA and CA15.3 (96% and 97% respectively). With ≥ 15 U/mL MCA cut-off, MCA sensitivity increased from 53% to 58% after its association with CA15.3. Sensitivity of CEA-TPA-CA15.3 panel was 74% (14 of 19 recurrences). Eight of the 14 recurrences early detected with CEA-TPA-CA15.3 presented as a single lesion (oligometastatic disease) (5) or were confined to bony skeleton (3) (26% and 16% respectively of the 19 relapses). With ≥ 11 U/mL MCA cut-off, MCA-CA15.3 association showed higher sensitivity but lower specificity, accuracy and positive predictive value than the CEA-TPA-CA15.3 panel.

**Conclusion:**

At both the evaluated cut-off values serum MCA sensitivity is higher than that of CEA, TPA or CA15.3 but its specificity is similar to or lower than that of TPA. Overall, CEA-TPA-CA15.3 panel is more accurate than MCA-CA15.3 association and can "early" detect a few relapsed patients with limited metastatic disease and more favourable prognosis. These findings further support the need for prospective randomised clinical trial to assess whether an intensive post-operative follow-up with an appropriate use of serum tumour markers can significantly improve clinical outcome of early detected relapsing patients.

## Background

In breast cancer patients, current guidelines post-operatively recommend mammography at regular intervals and not routine use of any instrumental or laboratory test for early detection of relapse and monitoring of metastatic disease. In fact, in randomised trials and meta-analysis intensive post-operative follow-up has been shown to be useful only in early diagnosis but not in improving clinical outcome and/or quality of life [1-4]. Nevertheless, in these trials clinical-instrumental was compared with clinical only follow-up and neither any serum tumour marker panel nor appropriate criteria for its use were adopted. Different studies appropriately using serum tumour markers within an intensive post-operative follow-up showed that in many relapsing patients clinical-instrumental diagnosis was anticipated and that this anticipation permitted an earlier treatment which significantly prolonged disease-free survival (DFS) and/or overall survival (OS) [5-8]. Moreover we showed that this type of follow-up strongly reduced need for conventional radiological examinations [9-11]. Therefore, unlike current guidelines, it is routine practice in our center to carry out an intensive post-operative follow-up of breast cancer patients using both serum tumour markers and imaging techniques.

Carcinoembryonic (CEA) and breast cancer associated 115D8/DF3 (CA15.3) antigens are the serum tumour markers commonly used for post-operative monitoring of breast cancer [1] although many other tumour markers have been investigated [12-17]. We reported similar sensitivity for TPA and CA15.3, which, however, is higher than that of CEA. TPA showed much lower specificity than CA15.3 and CEA. The association of these three markers increased sensitivity with a slight decrease of specificity [4-6,18].

CA15.3 is one of the mucin-like biomarkers which also recently have been reported among the most useful markers to detect and monitor metastatic breast cancer [8,19]. The mucin-like carcinoma associated antigen (MCA) is another widely used test to assay MUC-1. When it has been used alone, high sensitivity and specificity have been reported [17,20,21]. This suggests the association with CA15.3. However, different cut-off values have been reported for MCA [17,20]. Besides conflicting data have been found both as to MCA sensitivity and specificity compared to CA15.3 [22-25] and the MCA-CA15.3 usefulness [14,15,22,26,27]. Finally, as far as we know, no previous study compared MCA-CA15.3 to CEA-TPA-CA15.3 association. Therefore, in this study we compared sensitivity and specificity of MCA (with two commonly used cut-off values: ≥ 11 and ≥ 15 UI/mL) with that of CEA, CA15.3, TPA for early detection of relapse. Moreover, also we compared the diagnostic accuracy and predictive value of the MCA-CA15.3 association to that of CEA-TPA-CA15.3 panel.

## Methods

### Patients' follow-up

From March 2000 to September 2003, 289 breast cancer patients aged 27 to 80 years (median 51) were submitted to an intensive post-operative follow-up with serial serum determination of CEA, CA15.3, TPA and MCA. At entry 268 patients (93%) were disease-free (M0) and 21 (7%) showed distant metastases (M1). At the post-operative histology 83 (31%) of the 268 disease-free patients were N+ while the 185 remaining were N-. Premenopausals were 162 (56%) of all the 289 studied patients. Soon after primary surgery, all estrogen (ER) and/or progesterone receptor(PgR) positive patients received hormone therapy. Moreover, all N+ and 129 N- disease-free patients, consistent with the current international guidelines, received adjuvant chemotherapy. As to the interval time of post-operative monitoring, patients were divided into 2 groups: at low and at intermediate-high risk of recurrence according to whether they were N-PgR+ or N+ and/or PgR- (N+ PgR+, N+ PgR-, N- PgR-) respectively. Axillary lymph-nodes (N+/N-) [28] and progesterone (PgR+/PgR-) status [29,30] were used to divide patients into two different risk groups as they are commonly reported among the principal prognostic factors for relapse. The 126 low risk patients underwent control visits every 6 months and the remaining 142 with intermediate-high risk of recurrence every 4 months. Post-operative follow-up was 16 ± 8 months (m ± sd ; range 6–37 months). At each control visit, history, routine lab and serum CEA, CA15.3, TPA and MCA measurement were carried out.

Baseline Sx-ray was performed to identify benign lesions due to inflammatory and/or degenerative disease. As to other conventional instrumental examinations, bone scintigraphy (BS) and liver echography (LE) have been reported to be more accurate than chest x-ray (CXR) to "early" detect recurrences [7,9,10,16,31,32]. Therefore, BS and LE were serially performed every 24–30 months and CXR at more prolonged intervals (mean value 42 months).

The reason for serial BS, LE and CXR examinations, even if more prolonged, was to detect asymptomatic relapses falsely negative with serum tumour markers, which as we have previously reported [4,7,9-11] are about 15–25% using CEA-TPA-CA15.3 tumour marker panel.

Patients suspected of relapse with CEA-TPA-CA15.3 tumour marker panel immediately underwent the standard radiological examinations (BS, LE and CXR). If these examinations were pathological or equivocal, patients immediately were selected to be further investigated as follows. All hot spots on the bone scintigraphy with an equivocal interpretation were examined by computed tomography and/or magnetic resonance imaging (MRI). The lesions that were considered equivocal by conventional chest x-ray were clarified by computed tomography or bronchoscopy and cytologic study. The lesions felt to be equivocal at liver echography were clarified by computed tomography or fine needle aspiration cytology guided by liver echography when possible. The patients with equivocal standard radiological examinations and concomitantly not suspected of relapse with the CEA-TPA-CA15.3 tumour marker panel, were regularly followed-up as above described.

All patients gave their consent to be post-operatively monitored with all instrumental and laboratory examinations described in the paper.

### Tumour markers

Serum CEA, TPA, CA15.3 and MCA concentrations were measured in fasting patients by commercial immunoenzimatic assays: Abbott, Rome (Italy) for CEA and CA15.3; DRG, Marburg (Germany) for TPA; Roche Diagnostics, Manheim (Germany) for MCA. The within and between assay coefficients of variation for CEA, CA15.3, TPA and MCA were all less than 5% and 6% respectively. Serum levels > 7 ng/mL, > 95 U/L and > 32 U/mL were considered to be elevated for CEA, TPA and CA15.3 respectively; for MCA, ≥ 11 and ≥ 15 U/mL cut-off values were considered. We identified the causes of false positive tumour marker increase [17]. As previously described [4,9,10], a dynamic evaluation of tumour markers was made and in cases of a high tumour marker value a further blood sample was drawn two weeks to a month after the previous elevated value. If the re-measured tumour marker value had decreased to a normal value, the initial elevated value was considered to be an isolated elevated value (IEV). The elevated tumour marker was considered to be progressive (PI) when it was 30%, or more, higher in the sample which followed the initial elevated value. Otherwise, two equally high values were regarded to be a constant elevation (CE). Only CE and/or PI were considered a significant tumour marker increase.

As previously reported [9-11] in our clinical practice only patients with CE or PI in one or more tumour markers, clearly unexplained by any other condition, are considered suspected of tumour relapse.

Tumour marker lead time was the time from the suspicion of relapse with serum tumour marker to confirmation of relapse by radiological examinations. When a clinically disease-free patient was suspected of relapse by re-testing of tumour markers at the regular control visit, 15 to 30 days were necessary to carry out the common (bone scintigraphy, liver echography, chest x-ray) and in case of their equivocal result, more accurate radiological examinations (CT, MRI) to confirm or rule out the suspicion. Radiological investigations performed during this 15 to 30 days interval time and confirming the initial suspicion by tumour markers were considered as they had been performed at the same time of tumour marker re-testing; therefore in this case tumour marker lead time was computed as zero. When a patient became suspected of metastases by symptoms before the routine testing of serum tumour markers (i.e. in the interval between two regular control visits), at this time immediately the entire planned procedure was carried out to confirm or rule out the suspicion. Again, as above mentioned, the time necessary for the entire procedure took about 15 to 30 days and this interval time was not considered for the calculation of the tumour marker lead time. In fact, when suspicion of metastases was confirmed by radiological examinations and not by tumour marker panel, tumour marker panel was considered falsely negative. When suspicion of metastases was confirmed by radiological examinations and by tumour marker panel as well, the tumour marker panel lead time was considered zero if symptoms suspicious of metastases had appeared at the same time the entire procedure for confirmation was started; if symptoms suspicious of metastases had previously appeared, clinical symptoms only were considered the first signal of relapse and tumour marker panel was considered falsely negative.

### Statistical analysis

Sensitivity was defined as TP/(TP+FN)% specificity as TN/(TN+FP)%, accuracy as TN+TP/(TN+FN+TP+FP), positive predictive value as TP/(TP+FP), negative predictive value as TN/(TN+FN), where FP = false positive, FN = false negative, TP = true positive, TN = true negative. Two analyses were performed. In a first analysis only dynamic evaluation of tumour markers was taken into account without considering clinical and laboratory data. In this analysis tumour marker assay with CE or PI in one or more markers was defined as a probably positive test. In a further analysis dynamic evaluation of tumour markers was considered with an accurate clinical history and laboratory examinations. In this analysis tumour marker assay with CE or PI in one or more markers, unexplained by any concomitant transient or chronic benign pathology, that is for unknown reasons, was defined as a probably positive test. In both analyses tumour marker assay with normal or IEV was defined as a probably negative test. In the further analysis also CE or PI in one or more markers likely due to a clear concomitant benign pathology was defined as a probably negative test.

A probably positive test, according to whether it was or was not confirmed by monitoring to death or by the development of a definite clinical-imaging course after the initial result, was defined as true or false positive respectively. A probably negative test, according to whether it was or was not confirmed by monitoring by at least one year without any clinical-imaging sign of relapse, was defined as true or false negative.

Because one of 18 relapsed patients recurred twice during the follow-up (see the Result section), statistical analysis was performed considering 19 relapses.

## Results

During the post-operative follow-up distant metastases occurred in 18 (6.7%) of the 268 disease-free patients. Nine (50%) of these 18 patients were postmenopausals. One of them recurred twice. In this patient multiple bone and liver metastases were found 9 months after she had been rendered disease-free with surgical removal of a single secondary liver nodule. Therefore, as total, there were 19 relapses. The organs initially involved in the relapse were: bone (8), viscera (8), soft tissue (1), bone and viscera (2). The number of the lesions was: < 3, > 3 < 10 and > 10 in 8, 6 and 5 relapses respectively.

Sensitivity of each tumour marker: CEA,TPA,CA15.3 and MCA (≥ 11 or ≥ 15 U/mL cut-off value) (Table 1)

MCA cut-off value ≥ 11 U/mL. MCA, CEA, CA15.3 and TPA were the first finding in 2 to 13 relapses. In 6 relapses for MCA and in 1 for CEA the tumour marker increase was the only sign. In 7 relapses for MCA, in 1 for CEA and in all 5 and 6 relapses for TPA and CA15.3 respectively the tumour marker increase was concomitant with the increase of other markers and/or with clinical or instrumental findings. The mean lead time from the tumour marker increase to the appearance of clinical and/or instrumental signs of the relapse ranged from 2 ± 2.8 for CEA to 7.1 ± 6.8 months for MCA. BS alone or combined with tumour markers (one or more) was the first finding of relapse more frequently than LE or clinical symptoms (4 vs 1 and 2 relapses respectively). In these instances the mean lead time from the appearance of clinical and/or instrumental signs to tumour marker increase ranged from 0 for LE to 3 ± 4.2 months for clinical symptoms.

MCA cut-off value ≥ 15 U/mL. MCA, CEA, TPA and CA15.3 were the first finding in 3 to 10 relapses. In 1 relapse for MCA and for CEA the tumour marker increase was the only sign. In 9 relapses for MCA, in 2 for CEA and in all 8 and 6 relapses for TPA and CA15.3 respectively the tumour marker increase was concomitant with the increase of other markers and/or with clinical or instrumental findings. The mean lead time from tumour marker increase to the appearance of clinical and/or instrumental signs of the relapse ranged from 2.9 ± 4 for TPA to 7.3 ± 9.4 months for CEA. Again, BS alone or combined with tumour marker increase was the first finding of relapse more frequently than LE or clinical symptoms (4 vs 2 and 3 relapses respectively). In these instances the mean lead time from the appearance of clinical and/or instrumental signs to tumour marker increase ranged from 2 ± 3.5 for clinical symptoms to 5.5 ± 7.8 months for LE.

Sensitivity of different tumour marker associations: MCA (≥ 11 or ≥ 15 U/mL cut-off values) – CA15.3 and CEA-TPA-CA15.3 panels (Table 2)

MCA cut-off value ≥ 11 U/mL. MCA-CA15.3 and CEA-TPA-CA15.3 panels were the first finding of 13 and 10 relapses respectively. In 6 relapses for the former association and in 2 for the latter the tumour marker increase was the only sign while in the remaining it was the first finding of relapse concomitant with clinical and/or instrumental examinations. BS alone or combined with tumour marker increase was the first finding in 4 relapses, while it occurred in 1 relapse for LE and in 2 relapses for clinical symptoms. In these instances the mean lead time from the appearance of clinical and/or instrumental signs to tumour marker increase ranged from 0 for LE to 3 ± 4.2 months for clinical symptoms.

MCA cut-off value ≥ 15 U/mL. CEA-TPA-CA15.3 panel and MCA-CA15.3 association were the first finding of 14 and 11 relapses respectively. In 2 relapses for the former association and in 1 relapse for the latter the tumour marker increase was the only sign while in the remaining it was the first finding of relapse concomitant with clinical and/or instrumental examinations. Eight of the 14 recurrences "early" detected using CEA-TPA-CA15.3 tumour marker panel presented as a single lesion (5) or confined to bony skeleton (3). Three of the 5 recurrences that presented as single lesions involved bony skeleton and the 2 remaining liver. Therefore, "early" detected recurrences that presented as a single lesion or limited to bony skeleton were 26% and 16% respectively of the 19 relapses. BS alone or combined with tumour marker increase was the first finding in 4 relapses, while it occurred in 2 relapses for LE and in 3 relapses for clinical symptoms. In these instances the mean lead time from the appearance of clinical and/or instrumental signs to tumour marker increase ranged from 1.5 ± 3 for BS to 5.5 ± 7.8 months for LE.

With both MCA cut-off values, no significant difference occurred between the lead time of MCA-CA15.3 and CEA-TPA-CA15.3 panels (p n.s., unpaired t test).

Specificity of each tumour marker: CEA, TPA, CA15.3, MCA

CE and/or PI occurred in 11 patients for CEA and in 8 patients for CA15.3. Diabetes and/or hepatic steatosis (5 patients), smoking (4 patients), miscellanea (1 patient) for CEA, chronic liver failure (2 patients), diabetes and/or hepatic steatosis (5 patients), hepatic cyst or angioma (1 patient) for CA15.3 were the concomitant conditions probably responsible for these two kinds of tumour marker increase. Significant increases for unknown reasons (false positives) occurred in no patient for CA15.3 and in 1 patient (0.4%) for CEA. CE and/or PI occurred in 69 patients for TPA and in 144 patients or in 68 patients for MCA with ≥ 11 U/mL or ≥ 15 U/mL cut-off value respectively. Chronic liver failure (7 patients), diabetes and/or hepatic steatosis (36 patients), acute inflammation of upper airways (6 patients) for TPA, chronic liver failure (6 patients), diabetes and/or hepatic steatosis (54 patients), acute joint inflammation (9 patients), acute inflammation of upper airways (9 patients), hepatic cyst and/or angioma (9 patients), miscellanea (7 patients) for MCA with ≥ 11 U/mL cut-off value, chronic liver failure (5 patients),diabetes and/or hepatic steatosis (23 patients), acute joint inflammation (5 patients), hepatic cyst and/or angioma (6 patients) for MCA with ≥ 15 U/mL cut-off value were the concomitant conditions more often probably responsible for these two different kinds of tumour marker increase. Significant increases for unknown reasons (falsely positives) were found in 10 (4%), 43 (17.2%) and 20 (8%) patients for TPA, MCA with ≥ 11 U/mL and MCA with ≥ 15 U/mL cut-off value respectively. Therefore, specificity was 100%, 100%, 96%, 83% and 92% for CEA, CA15.3, TPA and MCA (≥ 11 U/mL or ≥ 15 U/mL cut-off value) respectively, when an accurate history was taken into account. Without an accurate history, specificity was 96%, 97%, 72%, 42% and 73% for CEA, CA15.3, TPA and MCA (≥ 11 U/mL or ≥ 15 U/mL cut-off value) respectively.

Specificity of different tumour marker associations: MCA (≥ 11 U/mL or ≥ 15 U/mL cut-off value) – CA15.3 and CEA-TPA-CA15.3

CE and/or PI of CEA-TPA-CA15.3 panel occurred in 77 patients. Diabetes and/or hepatic steatosis (40 patients), chronic liver failure (7 patients), acute inflammation of upper airways (5 patients) were the concomitant conditions probably responsible for the tumour markers' increase.

When MCA cut-off value was ≥ 11 U/mL, CE and/or PI of MCA-CA15.3 association occurred in 151 patients. Diabetes and/or hepatic steatosis (58 patients), hepatic cyst and/or angioma (12 patients), acute joint inflammation (10 patients), acute inflammation of upper airways (9 patients) were the concomitant conditions more often probably responsible for these two different kinds of tumour marker increase.

When MCA cut-off value was ≥ 15 U/mL, CE and/or PI of MCA-CA15.3 association occurred in 72 patients. Diabetes and/or hepatic steatosis (24 patients), hepatic cyst and/or angioma (7 patients), chronic liver failure (6 patients) were the concomitant conditions more often probably responsible for these two different kinds of tumour marker increase. Significant increases for unknown reasons (falsely positives) occurred in 11 (4.4%), 41 (16.4%) and 19 (7.6%) patients for CEA-TPA-CA15.3 panel, MCA-CA15.3 association with cut-off value ≥ 11 U/mL and MCA-CA15.3 association with cut-off value ≥ 15 U/mL respectively.

Therefore, specificity was 96%, 84% and 92% for CEA-TPA-CA15.3 and MCA (≥ 11 U/mL or ≥ 15 U/mL cut-off value) – CA15.3 associations respectively, when an accurate history was taken into account. Without an accurate history, specificity was 69%, 40% and 71% for CEA-TPA-CA15.3 and MCA (≥ 11 U/mL or ≥ 15 U/mL cut-off value) – CA15.3 associations respectively.

Reliability of MCA (≥ 11 U/mL or ≥ 15 U/mL cut-off values) – CA15.3 and CEA-TPA-CA15.3 tumour marker panels with and without an accurate history (Table 3)

MCA cut-off value ≥ 11 U/mL. When dynamic evaluation of tumour marker associations was considered with an accurate history and laboratory examinations, specificity, accuracy and PPV of CEA-TPA-CA15.3 tumour marker panel were higher than the corresponding of MCA-CA15.3 association. The opposite occurred for sensitivity and NPV. When dynamic evaluation of tumour markers was considered alone, specificity, accuracy, NPV and PPV of CEA-TPA-CA15.3 tumour marker panel were higher than the corresponding of MCA-CA15.3 association. The opposite occurred for sensitivity.

MCA cut-off value ≥ 15 U/mL. When dynamic evaluation of tumour marker associations was considered with an accurate history and laboratory examinations, sensitivity, specificity, accuracy, NPV and PPV of CEA-TPA-CA15.3 tumour marker panel were higher than the corresponding of MCA-CA15.3 association. When dynamic evaluation of tumour markers was considered alone, sensitivity, accuracy, NPV and PPV of CEA-TPA-CA15.3 tumour marker panel were higher than the corresponding of MCA-CA15.3 association. The opposite occurred for specificity (Table 3).

## Discussion

MCA sensitivity for "early" detection of relapses, either with ≥ 11 or ≥ 15 UI/mL cut-off value, was higher than that of CEA or CA15.3 or TPA. With regard to CEA, TPA and CA15.3, CA15.3 (with MCA cut-off value ≥ 11 UI/mL) and TPA (with MCA cut-off value ≥ 15 UI/mL) were more sensitive than both remaining indicators. However, only with ≥ 11 U/mL MCA cut-off value, sensitivity increased slightly (from 53% to 58%) after the association of MCA with CA15.3, while as we [4,7,9-11] and others [22,26,33] previously reported, it occurred significantly when TPA was associated with CA15.3 and CEA (Tables 1, 2). In other studies a range of MCA sensitivity similar to CA15.3 and no significant increase in sensitivity when MCA was combined with CA15.3 were found [14,27,34,35]. These findings and our results suggest that, although MCA and CA15.3 recognise distinct epitopes on the same molecule [36], in metastatic breast cancer cells MCA expression almost completely overlaps that of CA15.3, while it partially occurs among CEA, TPA and CA15.3.

Total rate of significant increases of MCA was similar to or higher (≥ 11 or ≥ 15 UI/mL cut-off value respectively) than that of TPA and it was higher than that of CEA and CA15.3. Consequently, significant increases of MCA-CA15.3 association occurred more frequently (60% vs 31%) or similarly (29% vs 31%) to those of CEA-TPA-CA15.3 tumour marker panel. This finding does not confirm that MCA specificity is similar to or higher than that of CEA and CA15.3 [17,20-23]. Moreover, our results show that in non relapsed patients the aspecific reasons probably responsible for MCA increase are the same as for TPA. The addition, at each control visit, of an accurate history and laboratory examinations to the dynamic evaluation of tumour markers increased their specificity while sensitivity remained unchanged. In fact, in 8 (42%) of the 19 relapses, a concomitant benign pathology occurred. Nevertheless, in all of them CE and/or PI in one or more markers could be referred to a pending relapse rather than to the concomitant benign pathology. Conversely, among the non relapsed patients those falsely suspected with all evaluated tumour markers particularly MCA, TPA and their combinations strongly decreased. In fact, CE and/or PI, unexplained by a clear concomitant benign pathology, ranged from 0% for CA15.3 to 17% for MCA with ≥ 11 cut-off value. When MCA cut-off value was ≥ 11 UI/mL, MCA-CA15.3 association showed higher sensitivity and NPV but lower specificity, accuracy and PPV than CEA-TPA-CA15.3 panel. When MCA cut-off value was ≥ 15 UI/mL, sensitivity, specificity, accuracy, NPV and PPV of CEA-TPA-CA15.3 panel were higher than those of MCA-CA15.3 association (Table 3).

Being confined to bony skeleton is considered a favourable prognostic factor for metastatic disease [37,38]. Also it has been shown that a single lesion, i.e. minimal metastatic disease called "stage IV oligometastatic disease", amenable to local therapy (surgery and/or radiation) followed by high dose chemotherapy is considered another different favourable condition [39,40]. In these metastatic patients with oligometastatic disease or disease limited to bony skeleton, median overall survival 2–3 times longer than in general metastatic population is expected. In a general metastatic population at the presentation bony skeleton as dominant site and oligometastatic disease have been reported to involve about 15% [41-43] and 5–10% [38,44,45] of patients respectively. Therefore, in this study a post-operative follow-up with an appropriate use of CEA-TPA-CA15.3 tumour marker panel also "early" detected a relatively high percentage of relapsed patients with limited metastatic disease and more favourable prognosis.

## Conclusion

In conclusion, data from this study point out that at both the evaluated cut-off values serum MCA sensitivity is higher than that of CEA, TPA and CA15.3. However, MCA specificity is the lowest or similar to that of TPA and they are both much lower than those of CEA and CA15.3. Despite a higher sensitivity, this low specificity represents an important limitation for a meaningful clinical applicability of MCA and TPA as single marker. Furthermore they suggest that overall CEA-TPA-CA15.3 panel is more suitable than MCA-CA15.3 association for an intensive post-operative follow-up of breast cancer patients with tumour markers and that an intensive post-operative follow-up with CEA-TPA-CA15.3 panel can early detect relapsing patients with more favourable prognosis. These findings further urge the need for randomised clinical trial to assess whether an "early signalling" and treatment of distant metastases with an appropriate use of serum tumour markers also can significantly improve OS. Moreover, a detailed cost-efficacy ratio analysis from these trials will permit to draw definite conclusions about the usefulness of serum tumour markers for a routine use.

## Abbreviations

CEA: Carcinoembryonic antigen

TPA: tissue polypeptide antigen

CA15.3: breast cancer associated 115D8/DF3 antigen

MCA: mucin-like carcinoma associated antigen

DFS: disease-free survival

OS: overall survival

N: axillary lymph-node involvement

PgR: progesterone receptor

Sx-ray: skeletal x-ray

BS: Bone scintigraphy BS

LE: liver echography

CXR: chest x-ray

IEV: isolated elevated value

CE: constant elevation

PI: progressive increase

FP: false positive

FN: false negative

TP: true positive

TN: true negative.

NPV: negative predictive value

PPV: positive predictive value

## Competing interests

The author(s) declare that they have no competing interests.

## Authors' contributions

AN was responsible for study design, data analysis and preparation of manuscript; GT, AC, PF, LA, MC, PM for data analysis and preparation of manuscript; MRM for laboratory data and data analysis.

## Pre-publication history

The pre-publication history for this paper can be accessed here:



## Figures and Tables

**Table 1:** Sensitivity of MCA (*≥11 or **≥15 U/mL cut-off values), CEA, TPA, CA15.3 in the early detection of 19 relapses during an intensive post-operative follow-up with tumour markers.

	CE or PI of tumour markers as the initial pathological finding of relapse				
	
Tumour marker type	^a^Relapses (total, n)	Sensitivity (%)	Tumour marker increase		Lead time (months) m + sd (range)
			type	n	
	
*MCA	13	68	CE PI	8 5	7.1 ± 6.8 (0–22)
CEA	2	10	CE PI	0 2	2.0 ± 2.8 (0–4)
TPA	5	26	CE PI	3 2	3.2 ± 4.4 (0–9)
CA15.3	6	32	CE PI	5 1	4.7 ± 5.8 (0–14)
**MCA	10	53	CE PI	7 3	6.5 ± 6.8 (0–18)
CEA	3	16	PI	3	7.3 ± 9.4 (0–18)
TPA	8	42	CE PI	6 2	2.9 ± 4.0 (0–9)
CA15.3	6	32	CE PI	5 1	4.7 ± 5.8 (0–14)

^a^Total number of patients = 18; one patient after radical removal by surgery of a single metastatic lesion relapsed (also see Result section). Therefore, percentages are calculated on 19 relapses; CE = constant elevation; PI = progressive increase.

**Table 2:** Sensitivity of MCA (*≥11 or **≥15 U/mL cut-off values)-CA15.3 and CEA-TPA-CA15.3 panels in the early detection of 19 relapses during an intensive post-operative follow-up with tumour markers.

	CE or PI of tumour markers as the initial pathological finding of relapse				
	
Tumour marker association	^a^Relapses (total, n)	Sensitivity (%)	Tumour marker increase		Lead time (months) m + sd (range)
			type	n	
	
*MCA-CA15.3	13	68	CE PI	8 5	7.1 ± 6.8 (0–22)
CEA-TPA-CA15.3	10	53	CE PI	5 5	3.9 ± 4.9 (0–14)
**MCA-CA15.3	11	58	CE PI	8 3	5.9 ± 6.7 (0–18)
CEA-TPA-CA15.3	14	74	CE PI	8 6	4.6 ± 5.9 (0–18)

^a^Total number of patients = 18; one patient after radical removal by surgery of a single metastatic lesion relapsed (also see Result section). Therefore, percentages are calculated on 19 relapses; CE = constant elevation; PI = progressive increase.

**Table 3:** Comparison between reliability of MCA (*≥11 U/mL or **≥15 U/mL cut-off values) – CA15.3 and CEA-TPA-CA15.3 associations with or without an accurate history during intensive post-operative follow-up with tumour markers of 289 breast cancer patients.

	Tumour marker panel			
	
Reliability	CEA-TPA-CA15.3	*MCA-CA15.3	CEA-TPA-CA15.3	**MCA-CA15.3
	
Sensitivity %	53 (53)	68 (68)	74 (74)	58 (58)
Specificity %	96 (69)	84 (40)	96 (69)	92 (71)
Accuracy %	92.5 (68)	82.5 (42)	94 (69.5)	90 (41)
NPV %	96 (95)	97 (94)	98 (97)	97 (92)
PPV%	48 (11)	24 (8)	56 (15)	37 (7)

Numbers into brackets refer to dynamic evaluation of tumour markers without an accurate history and laboratory examinations.
